# Augmented reality for dental implantology: a pilot clinical report of two cases

**DOI:** 10.1186/s12903-019-0853-y

**Published:** 2019-07-19

**Authors:** Gerardo Pellegrino, Carlo Mangano, Roberto Mangano, Agnese Ferri, Valerio Taraschi, Claudio Marchetti

**Affiliations:** 1 Oral and Maxillofacial Surgery Unit, DIBINEM, University of Bologna, 125, Via San Vitale 59, 40125 Bologna, Italy; 2 Digital Dentistry Section, University San Raffaele, Milan, Italy; 3 Fifthingenium, Milan, Italy; 4 University of Technology - Sydney, School of Life Sciences, Sydney, Australia; 5 Chief of Oral and Maxillofacial Surgery Unit, DIBINEM, University of Bologna, Bologna, Italy

**Keywords:** Computer-assisted surgery, Image-guided surgery, Implantology, Navigation system, Real-time tracking, Implant placement accuracy

## Abstract

**Background:**

Despite the limited number of articles dedicated to its use, augmented reality (AR) is an emerging technology that has shown to have increasing applications in multiple different medical sectors. These include, but are not limited to, the Maxillo-facial and Dentistry disciplines of medicine. In these medical specialties, the focus of AR technology is to achieve a more visible surgical field during an operation. Currently, this goal is brought about by an accurate display of either static or dynamic diagnostic images via the use of a visor or specific glasses.

The objective of this study is to evaluate the feasibility of using a virtual display for dynamic navigation via AR. The secondary outcome is to evaluate if the use of this technology could affect the accuracy of dynamic navigation.

**Case presentation:**

Two patients, both needing implant rehabilitation in the upper premolar area, were treated with flapless surgery. Prior to the procedure itself, the position of the implant was virtually planned and placed for each of the patients using their previous scans. This placement preparation contributed to a dynamic navigation system that was displayed on AR glasses. This, in turn, allowed for the use of a computer-aided/image-guided procedure to occur. Dedicated software for surface superimposition was then used to match the planned position of the implant and the real one obtained from the postoperative scan. Accuracies, using this procedure were evaluated by way of measuring the deviation between real and planned positions of the implants. For both surgeries it was possible to proceed using the AR technology as planned. The deviations for the first implant were 0.53 mm at the entry point and 0.50 mm at the apical point and for the second implant were 0.46 mm at the entry point and 0.48 mm at the apical point. The angular deviations were respectively 3.05° and 2.19°.

**Conclusions:**

From the results of this pilot study, it seems that AR can be useful in dental implantology for displaying dynamic navigation systems. While this technology did not seem to noticeably affect the accuracy of the procedure, specific software applications should further optimize the results.

## Background

Computer-assisted procedures are becoming more and more integrated into different fields of dentistry [1]. This is particularly evident in the increasing use of processes such as 3D printing and CAD-CAM methods in the manufacturing of dental implantology. This has not only allowed for a more accurate and diverse manufacturing capability but also dramatically expands on the production surgical templates often made in-house.

Currently, the examination of static guided surgery as a means of creating surgical templates to accurately position implants is ample. The conclusion drawn from this research is that should the implant be inserted with a margin of error of approximately 1 mm, the implant rehabilitation process will be mostly successful [2]. However, the working time for planning and producing the surgical template do not encourage or justify an ordinary use of this method [3]. Another method for computer-assisted surgery in dental implantology is image-guided surgery through dynamic navigation. Such surgical techniques are already largely used in major Neurosurgery, Maxillo-facial surgery, ORL, and Orthopedic surgeries and is quickly becoming popular in Implantology. Some papers published in past years report on the comparable accuracy between dynamic and static surgical navigation [4–6]. It was shown that dynamic navigation could overcome some of the disadvantages associated with static guided surgery. These included reducing costs and time needed for the impression and laboratory procedures of a static guided system. Another advantage of a dynamic guided system could be the ability to have a direct view of the surgical field as well as the possibility to use standard drills which is optimal in a case of mouth opening reduction [7]. In addition to this, dynamic navigation allows for changes in implant planning to be made at the time of surgery. This level of flexibility is not offered by statically derived surgical guides as they are fixed and cannot be altered once they are planned and manufactured. Also, tight single-tooth edentulous ridge areas can be fully guided using dynamic guidance as a dynamic guide is not restricted by drill tube size (i.e. in the anterior mandibular incisor sites). Furthermore, implant size is not limited with dynamically guided systems, as they are with static guides and CBCT, planning and surgery can be achieved in a single day [1, 8, 9].

However, a possibly problematic disadvantage of a dynamic guided system is the need to simultaneously pay attention to the patient as well as the output from the navigation system display. This unfavourable feature is exacerbated in systems where the tracking device is positioned on the same mobile carriage as the navigation system display. This could cause difficulties in following the virtual procedure while also keeping sight of the surgical site itself [10]. Systems that use a mobile screen fixed near the patient’s head on the dental chair may address this issue as they limit the movement of the surgeon’s head and, therefore, their loss of sight of the surgical site [11].

The use of AR through specific glasses and an integrated screen is a fairly new trend in the field of medicine. This technology can allow the surgeon to visualize, in real-time, patient parameters, relevant x-rays, 3D reconstruction or a navigation system screen [12, 13]. This last item could significantly increase the use of dynamic navigation a process that has already been readily adopted in other major surgical disciplines. The use of these devices is currently under validation and only few publications are present in literature to date and even fewer papers investigate this technology in dentistry [10, 14]. The aim of our pilot study is to evaluate the feasibility of adopting AR as a means of facilitating the use of dynamic navigation for dental implantology. The secondary objective was to evaluate if the accuracy obtained with this innovative display device was maintained in the range already described in literature regarding dynamic navigation.

## Case presentation

Two patients were referred to the Oral and Maxillo-facial Unit of the Department of Biomedical and Neuromotor Sciences for implant supported prosthetic rehabilitation. Both patients were to be treated in the upper premolar area and were in good general health conditions and had no contra-indications to the implant surgery. The clinical procedures were carried out in accordance with national guidelines as well as with the Declaration of Helsinki.

### Navigation system setting

**Fig. 1:**
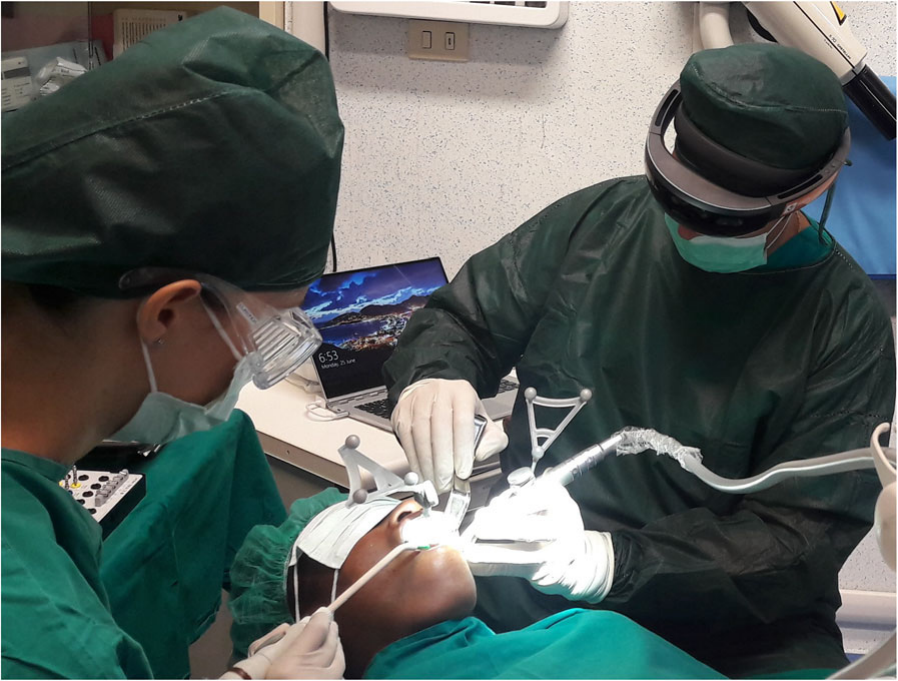
Overview of the Hololens glasses and navigation system reference tools during the surgery

After the filling of the appropriate consent documentation, both patients undertook a CBCT scan with the markers plate from the navigation system. These markers were positioned in situ as per protocol of using the navigation system ImplaNav (BresMedical, Sydney, Australia) which requires that the markers plate is fixed with a hard impression material (Ramitec, 3 M Espe, USA). After the scan, the markers plate was removed and replaced in the same position on the day of the surgery. The CBCT data was analyzed through the navigation system planning software and the position of two implants were virtually planned. At the time of the surgery the patient reference tool for the navigation system was fixed on the same support of the markers plate. Another reference tool was positioned and rigidly fixed on the implant drill handle. Then the calibration tool was connected to the handle and the drill axis was identified by the navigation system. The first lance drill was successively used to touch the fiducial markers on the markers plate to verify the patient position. After the calibration procedures, the navigation system was directly interfaced with the virtual reality glasses (Hololens, Microsoft, USA) through a wifi connection using a dedicated software created by Fifthingenium (Milan, Italy) (Fig. 1).

### Augmented reality glasses setting

Microsoft Hololens is an augmented reality headset which can be used to expand the limits of interaction between the virtual and the physical world. Hololens runs a custom Windows 10 version as its operating system. It also features Bluetooth and Wi-Fi connectivity and is powered by a Holographic Processing Unit HPU 1.0, 2GB RAM and 64GB of Solid State storage.It is also equipped with an Inertial Measurement Unit, four environment understanding cameras, mixed reality capture, four microphones, an ambient light sensor and two HD displays capable of automatic pupillary distance calibration.

The plethora of applications of the Hololens in industry is mainly attributed to its ability to create, manipulate and display holograms or virtual objects in the field of the user. Combined with the ability to recognize objects, rooms and environments through the use of AI and markers, the capabilities of the Hololens allows it to be useful in many industries including the Healthcare and Dental sector.

An application to use Hololens in the dental field was developed in order to visualize 2D/3D data (CBCTs, face scans, oral scans) while at the dental chair without forcing the practitioner to look at a specific monitor/computer. By controlling the device via only voice commands or simple gestures, the surgeon is able to maintain visual of the physical surgical site and avoiding contamination.

A system capable of mirroring the desktop of a computer on the Hololens was developed and coupled with the navigation system used for the surgery. Such system allows the doctor to avoid looking at the computer screen to receive guidance for the surgery. Instead, the doctor can visualize the system data, info, targets and positions by placing a virtual desktop near the patient’s face without being forced to look away from the patient’s mouth.

### Clinical procedure

**Fig. 2:**
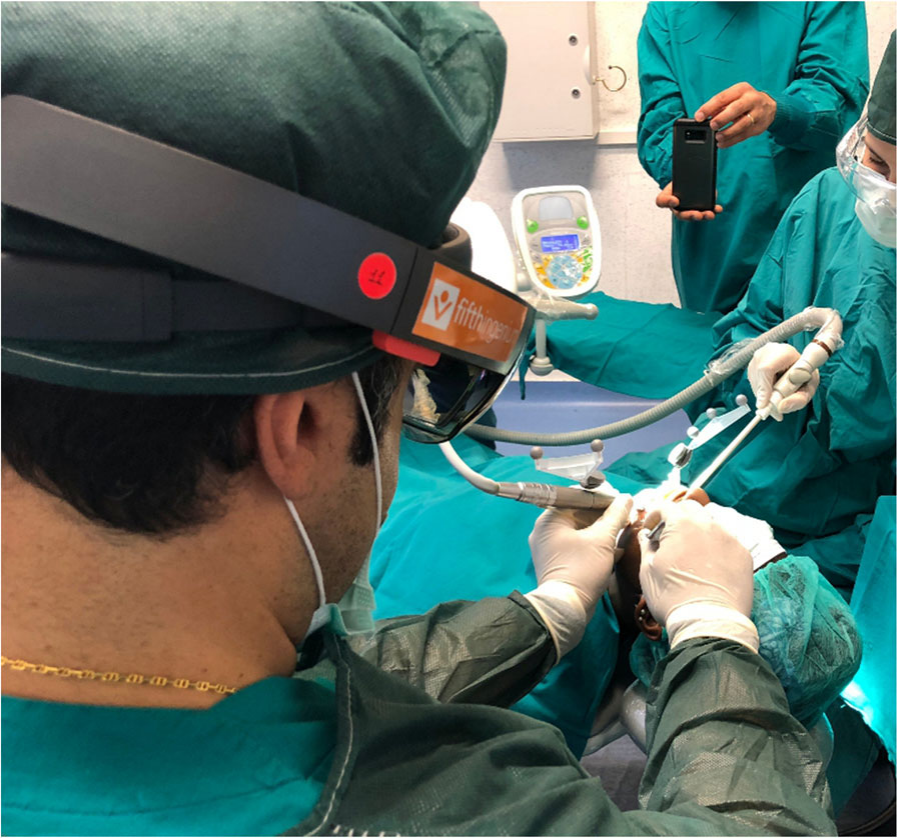
The external view of the surgeon during the surgical procedure

**Fig. 3:**
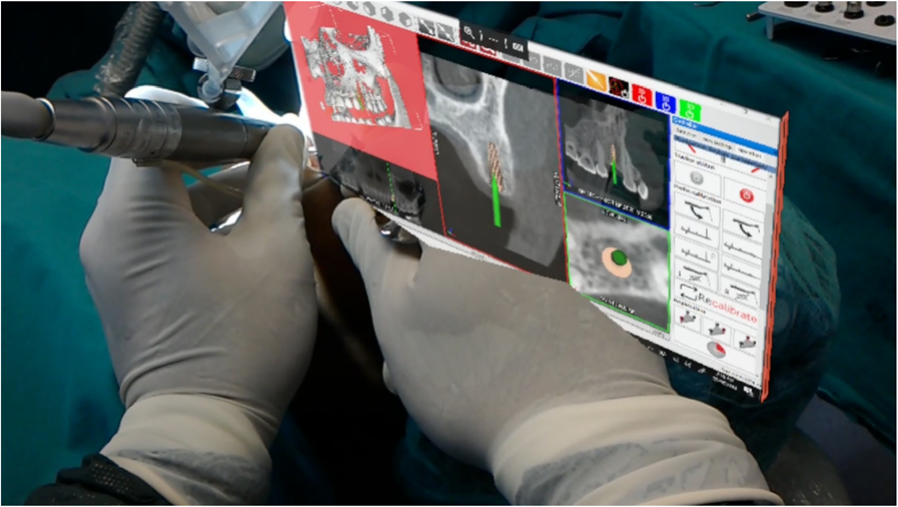
The view of the surgeon during the surgery wearing Hololens glasses

**Fig. 4:**
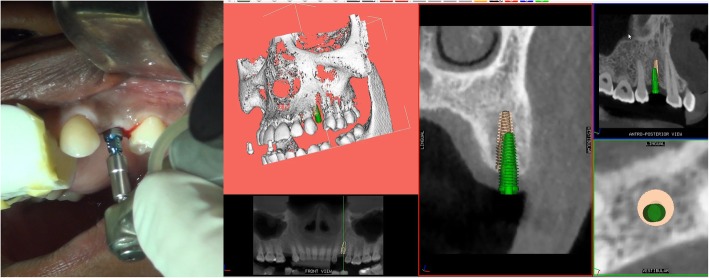
The real and the virtual implant position on the navigation system screen

**Fig. 5:**
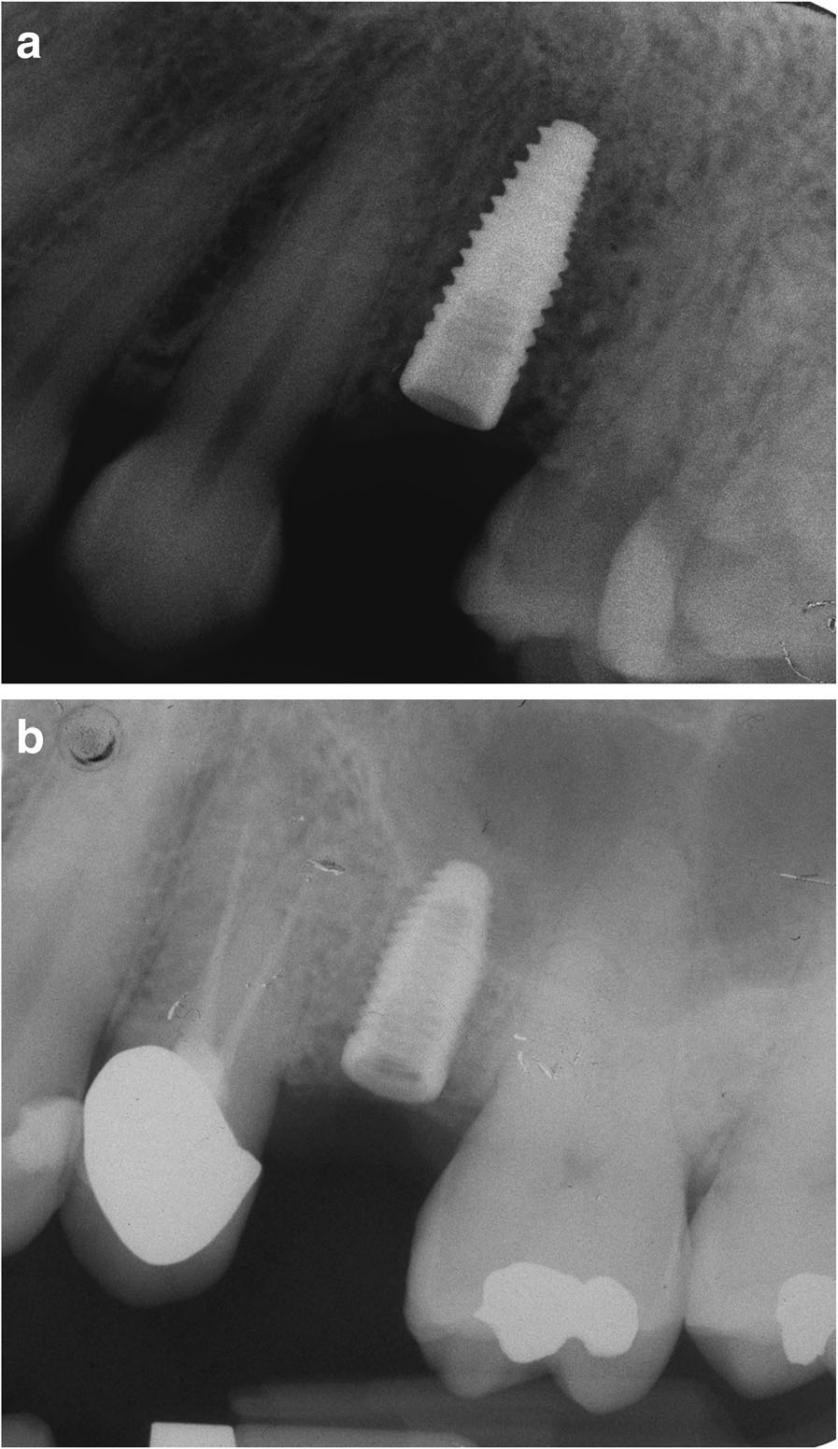
**a**, **b**: Postoperative radiographs

**Fig. 6:**
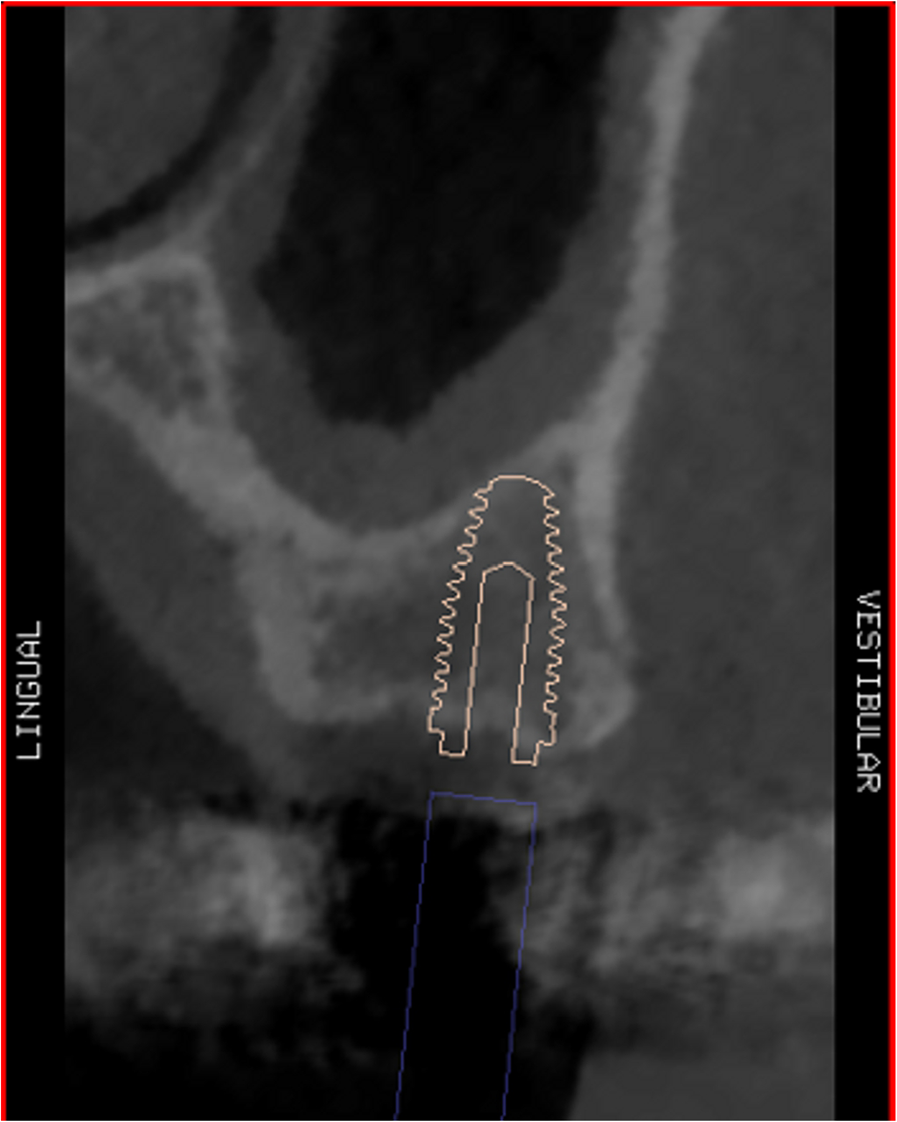
Implant position planned to be close to the maxillary sinus

**Fig. 7:**
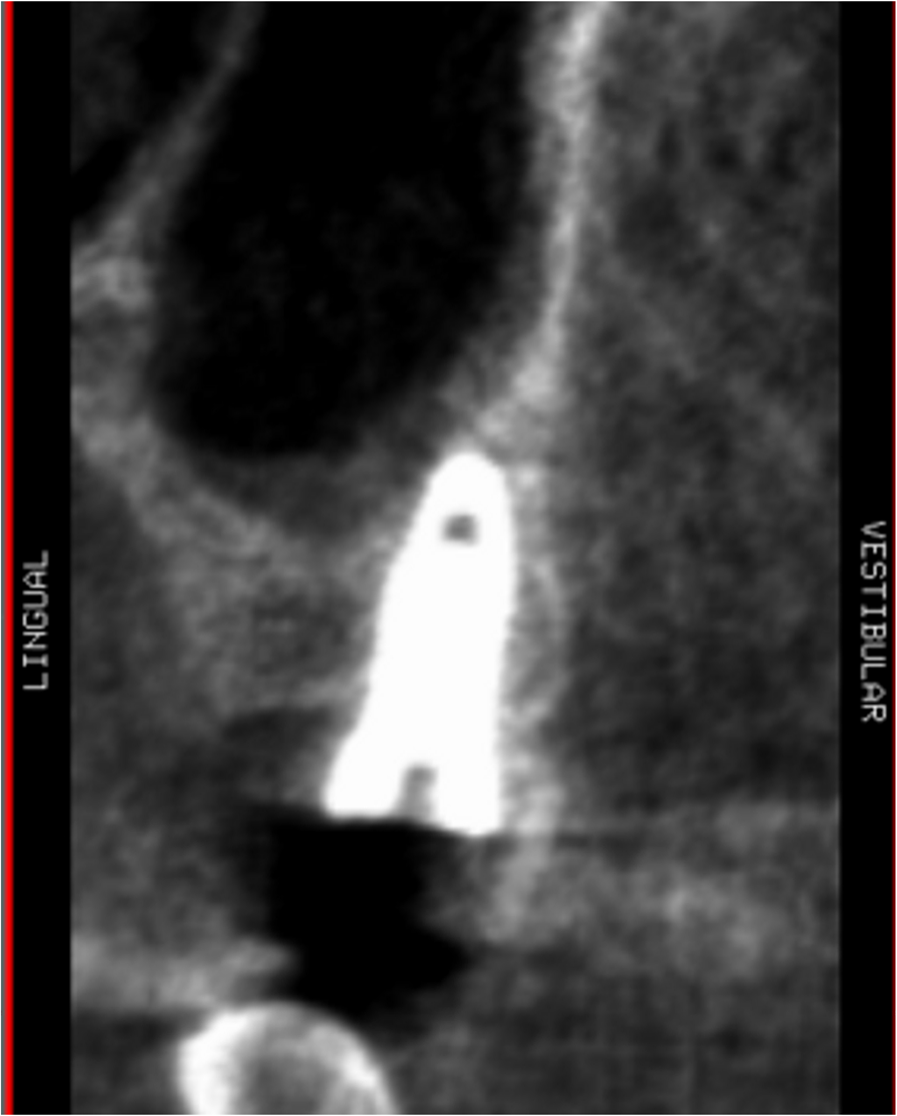
Postoperative implant position on CBCT

**Fig. 8:**
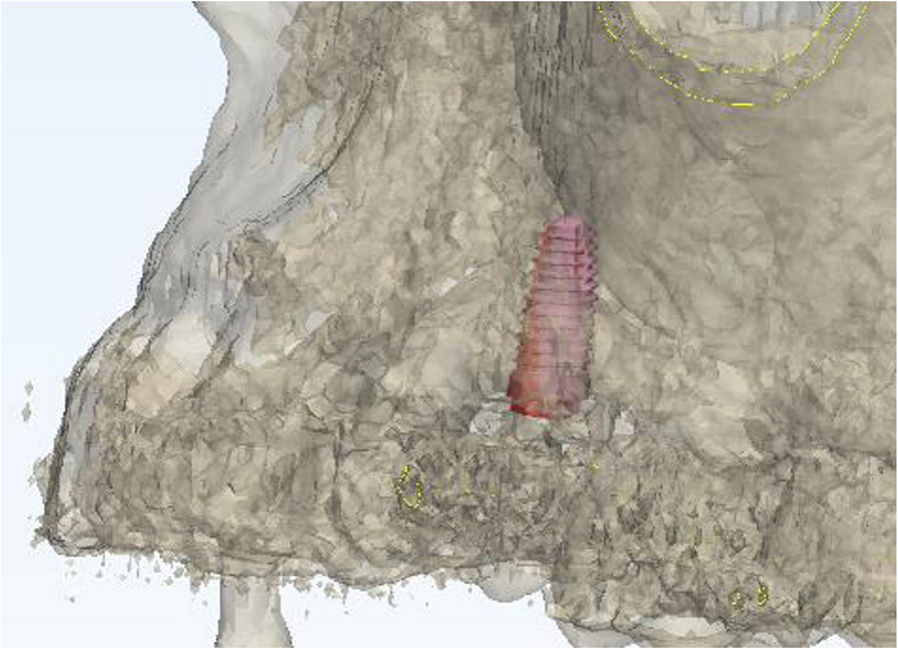
The two surfaces comprehending the teeth and the implants were compared via an N-point surface alignment of the teeth

**Fig. 9:**
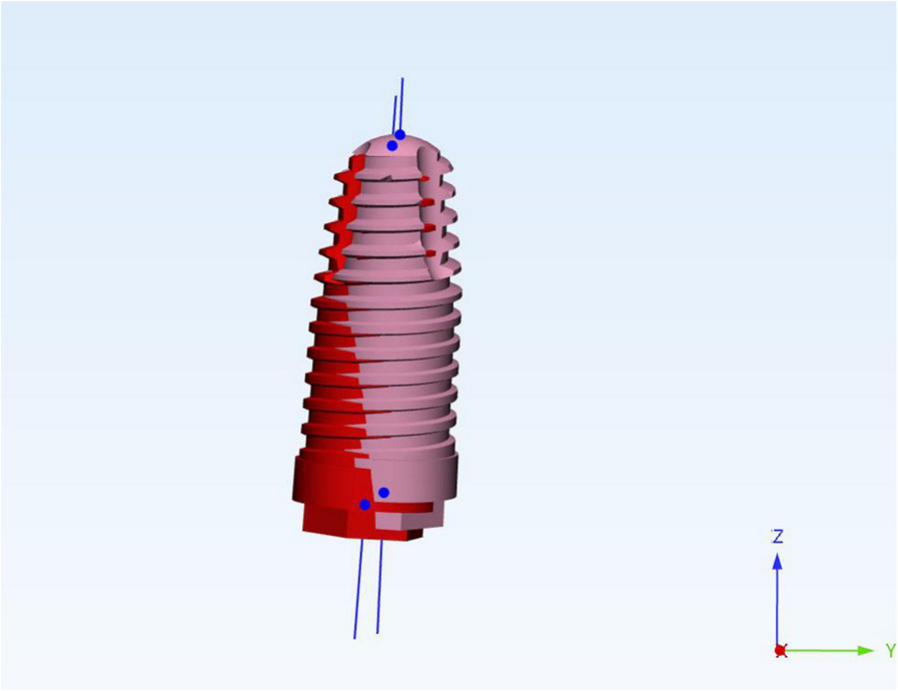
Superimposed view showing the correspondence between the planned and the real implant position

**Fig. 10:**
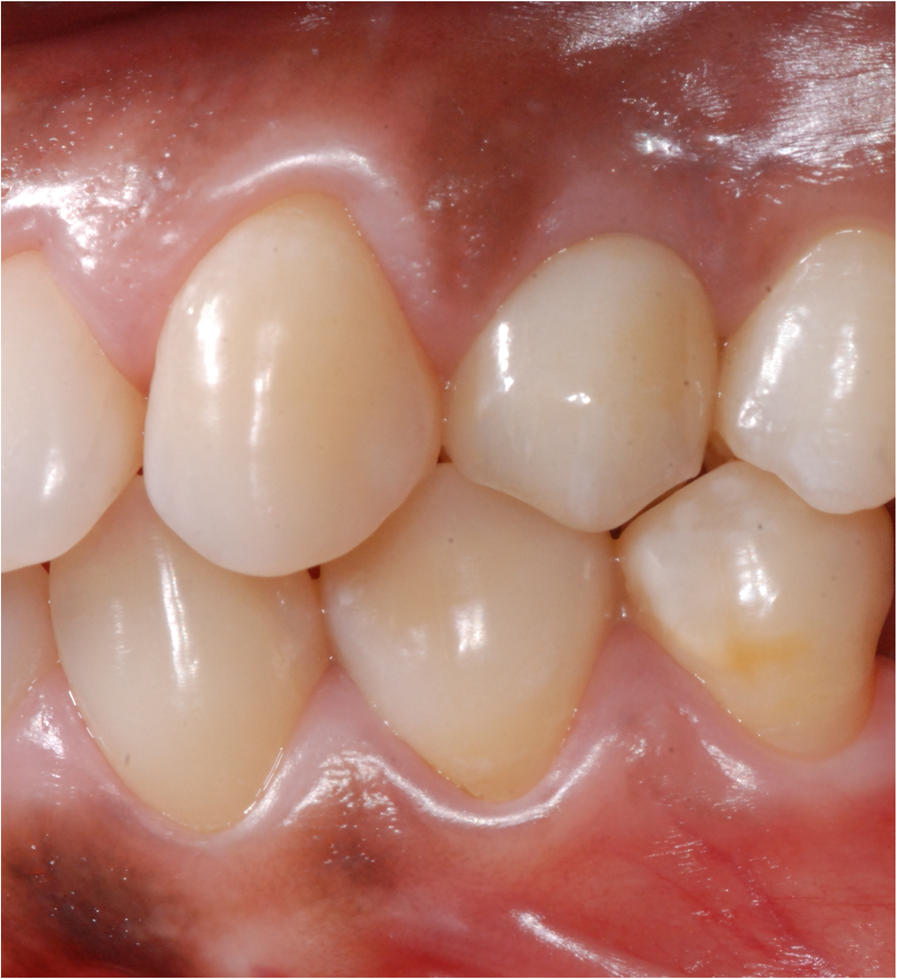
The prosthetic rehabilitation of one implant with a screw-retained crown

Using the Hololens glasses, the surgeon can contemporarily visualize the surgical field (Fig. 2) and the output of the navigation system screen. The virtual position and the trajectory of the drill into the bone, the implant planned position and the bone anatomy around the implant site were checked in real-time during the whole surgical procedure (Fig. 3). The navigation system software input can also be managed with Hololens through hand movements. Two implants were placed, one for each patient following the drill sequences provided by the implant company protocol. In one case a 3.8 × 9 mm (TTi, WinSix, Ancona, Italy) was positioned. In the other case a 4.1 × 11 mm (BL, Straumann, Switzerland). In both cases, a flapless surgery was carried out (Fig. 4). A postoperative radiograph was taken to evaluate the correct positioning of the implants (Fig. 5a, b). The healing abutments were fixed without any suture. In one case the implant position had been planned to be close to the maxillary sinus (Fig. 6) and postoperative CBCT was taken to verify if the goal had been reached (Fig. 7). After about 3 months, the contra-torque test was manually performed to verify the osseointegration status of the implants. Then through a scan of the abutment and the use of the intra-oral scanner, the implant position was digitally recorded concurrently with the bordering teeth. The virtual planned position of the implant and the adjacent teeth were exported from the planning module in the ImplaNav software. The two surfaces comprehending the teeth and the implants were compared via an N-point surface alignment of the teeth using Materialise 3-Matic (Materialise, Leuven, Belgium) (Fig. 8). The deviation between the planned implant position and the real one obtained by the scan were evaluated (Fig. 9). Both patients were rehabilitated with screw-retained crowns (Fig. 10).

## Results

In both cases it was possible to proceed with the navigation-aided implant placement with the Augmented Reality (AR) displaying in real-time a combination of surgical planning, real anatomy and the output from the navigation system (Fig. 3). The deviation between the planned and the real position of the implants resulted 0.53 mm at the entry point and 0.50 mm at the apical point for the first implant and 0.46 mm at the entry point and 0.48 mm at the apical point for the second one. The angular deviations were respectively 3.05° and 2.19° and the depth deviations were 0.26 mm and 0.37 mm.

## Discussion

Dynamic navigation is one of the two computer-guided surgery techniques used in implantology. Many authors reported relatively good results in terms of implant placement accuracy using different navigation systems [1, 15–17]. Block et al. [16] reported on the implant placement accuracy obtained by 3 surgeons using dynamic navigation to treat 100 partially edentulous patients. They reported a mean error of 0.87 ± 0.42 mm at the entry point, 1.56 ± 0.69 mm at the apex and 3.62° ±2.73° for angle deviations using dynamic navigation. Non-dynamically guided entry point deviations, apex deviations and angle discrepancies had corresponding mean values of 1.15 ± 0.59 mm, 2.51 ± 0.86 mm and 7.69° ± 4.92°. Stefanelli et al. [17], in a retrospective study on 231 implants reported an error of 0.71 ± 0.40 mm at coronal point, 1 ± 0.49 mm at apex and a mean angular error of 2.26 ± 1.62°. Although there are reported advantages using dynamic navigation, this method requires the surgeon to coordinate his view of the screen with the movements of his hands. The look out of the implant site with the rotation of the head for looking at the navigation system screen could represent a risk in case of accidental surgical instrument shifting or unexpected patient movement, especially in advanced implantology. The use of the augmented reality can overcome this drawback and also reduce operating time [10].

The categories of AR-guided surgery are grouped as follows: type I, involving the use of glasses or head-sets [12, 13]; type II, with digital data being projected on a half-silvered mirror [18]; type III, where the images are shown directly onto the patients; type IV, with the use of an external monitor [11]. In this study glasses have been used, allowing the contemporary projection of the patient’s anatomy and the virtual instruments near the surgical field. However, when a 3D virtual layer is displayed and laid over the real environment, there is often a discrepancy between the real image and the virtual image due to an overlay or positional error.

Augmented reality is employed in neurosurgery, laparoscopic digestive, laparoscopic thoracic, vascular, urological and gynecological laparoscopic and cardiac surgery. As per its application in maxillofacial surgery, most of the publications refer to its use in orthognathic surgery [13, 19, 20], traumatic surgery and reconstructive surgery [21–23]. In dentistry, AR is applied in orthodontics for guided bracket placement [24]. In endodontics it is applied to detect root canals and for educational and training purposes [25–27].

In implantology, few studies regarding the use of dynamic navigation, especially in vitro, have been published. Ewers et al. [14] reported a significant medical benefit for the patients when navigation and AR are used for implant placement. In an in-vitro study, Jiang et al. [10] demonstrated a smaller error in incisive and canine regions implant placement using AR associated with dynamic navigation as opposed to the use of 2D navigation methods. The surgery time was significantly shorter by using a combination of the two technologies. In the present study, a dynamic navigation system associated with the augmented reality was deployed. This technique allowed the surgeon to simultaneously having a view of the surgical field as well as the navigation system monitor displaying implant planning and virtual burs. By wearing glasses where the virtual image is projected near the surgical field, the surgeon could see the implant site without interference and without the risk of overlay errors.

The main limit of this technology, currently, is emanated by the sometimes inconvenient virtual window positioning and orientation together with the working distance of the glasses which could force the surgeon to operate in an uncomfortable position. Nevertheless, the cases reported were simple and these limitations did not affect the results. Despite this, a comfortable work position might become mandatory in advanced clinical cases [28, 29] in which this technology would prove to be beneficial. Other disadvantages could be considered the cost of the device, the time spent to set-up and the need to manage additional software for the AR. Possible setbacks could also occur from the device wireless connection and the battery charge although there were not reported in the present study. These problems could be solved by developing a dedicated software application for implantology and by upgrading the associated hardware.

As per the application in maxillofacial surgery of an AR technique displaying 3D images without the use of glasses, Suenaga et al. [30] reported a positional error of 0.77 ± 0,19 mm (range 0,45-1,34) and an angular error of 2°. Zhu et al. [12], however, reported a discrepancy of 0.96 ± 0,51 mm (range 0,55–2 mm). Most of the maximum overlay errors reported in literature are lower than 3 mm [11] with an exception for the research performed by Lin et al. [31], who reported a maximum error of 6.56 mm. The increase of accuracy, in addition to the lack of depth perception, is a problem the authors of these studies are working to address [32].

An in-vitro study by Lin et al. [31] showed good results in terms of implant placement accuracy using the drill-guides technique combined to AR. Katić et al. [33], by using an AR system in a pig cadaver experiment, reported a deviation of 1.1 mm and 2° between the planned implant and the positioned one. In the present case report, a less than 1 mm accuracy was achieved, comparable with the one reported in literature by only using the navigation system [1, 34]. This seems to indicate that AR does not affect the accuracy of the navigation procedure.

A touch-less interface for the navigation system software could also promote the use of this technology in the surgical theatre. By simplifying the procedures and reducing operative time, AR can proved to be an exceptional resource in dental implantology. This kind of technology could increase the use of dynamic navigation as it solves the problem of monitoring the screen and the patient simultaneously. The further development of AR could allow matching of the virtual with the real anatomy of the patient, a concept that is already under investigation for major surgery. At the moment, this is made difficult due to the need to follow the patient movement during the intervention usually carried out under local anesthesia.

## Conclusions

AR resulted to be quite useful in displaying dynamic navigation despite some software and hardware limits. The presence of the two environments in the AR does not seems to affect the accuracy of the surgical procedure. Specific software applications for navigation systems can further contribute to optimizing the results. Additional in vitro and clinical trials are required to validate the use of this new promising technology for dental implantology.

## Data Availability

All data generated or analyzed during this study are included in this published article.

## Abbreviations

AI: Artificial Intelligence
AR: Augmented Reality
CBCT: Cone-beam computerized tomography
mm: Millimeters
